# Relationship between Regional Distribution of Centenarians and Drinking Water Hardness in the Amami Islands, Kagoshima Prefecture, Japan

**DOI:** 10.3390/nu15071569

**Published:** 2023-03-24

**Authors:** Mai Suzuki, Siyuan Wu, Tomoki Ootawa, Henry Smith, Mitsuya Shiraishi, Atsushi Miyamoto, Yuki Matsuoka, Sawako Sawa, Mari Mori, Hideki Mori, Yukio Yamori

**Affiliations:** 1 Department of Veterinary Pharmacology, Joint Faculty of Veterinary Medicine, Kagoshima University, Kagoshima 890-0065, Japan; 2 Isen Town Office, Tokunoshima, Kagoshima 891-8293, Japan; 3 School of Health Studies, Tokai University, Hiratsuka 259-1292, Japan; 4 Institute for World Health Development, Mukogawa Women’s University, Nishinomiya 663-8143, Japan

**Keywords:** calcium, coral, limestone, longevity, magnesium, water hardness, water supply

## Abstract

People who drink naturally hardened water may experience longevity-enhancing effects. In this study, we investigated water hardness and longevity from both geological and epidemiological perspectives in Japan’s Amami islands, where drinking water is drawn from coralline or non-coralline bedrock. We investigated drinking water hardness, limestone bedrock occupancy, and the centenarian rate (number per 10,000 population) by municipality across four adjacent islands (Amami-Oshima (non-coralline), Tokunoshima, Okinoerabu, and Yoron (predominantly coralline)). Limestone was strongly correlated with water hardness (r = 0.99; *p* < 0.01), occupying more than 80% of the bedrock where the water was the hardest (Tokunoshima’s Isen municipality: 86.5%; Yoron: 82.9%) and being scarcely detectable in Amami-Oshima (0.0 to 0.2%), where the water was the least hard. The centenarian rate was also strongly correlated with water hardness (r = 0.84, *p* < 0.01), with the highest figures in Yoron (29.7) and Isen (29.2), and the lowest in Amami-Oshima (0.0 to 12.2). Therefore, we hypothesize a potentially beneficial effect of hard water on longevity when that water is drawn from coralline limestone. Water hardness is determined by the water content of calcium and magnesium and may plausibly influence life expectancy through a preventative effect against cardiovascular disease. Our findings are of interest to current debates about future global access to drinking water and its quality.

## 1. Introduction

The environment influences human longevity; this statement may appear axiomatic, but evaluating the influence of specific environmental factors can be challenging. Any factor can be difficult to disentangle from its confounders, and any evidence on such environmental influences needs to be fully contextualized biogeographically to be widely relevant.

Water hardness provides a case in point. People who drink naturally hardened water have long been believed to experience longevity-enhancing effects, and there is much anecdotal and scientific evidence to support this view, especially in regard to a protective effect against cardiovascular mortality. Even so, the relationship between the geochemistry of water and cardiovascular mortality has been described as “equivocal” [1], with the World Health Organization (WHO) stating that the evidence is debatable and does not yet prove causality [2]. In this study, we address the issue of water hardness and longevity from both geological and epidemiological perspectives.

Water is typically hardened by cations leeching from sedimentary layers as it percolates to the surface through the Earth’s crust. The predominant cations are calcium and magnesium, and their most common source is limestone (CaCO_3_). Water hardness is mainly calculated as the sum of calcium and magnesium ion concentrations [3]. These minerals may be better assimilated into the human body from drinking water than from foodstuffs [4,5], and researchers have investigated the beneficial effects of drinking water rich in calcium and magnesium in a number of studies since the 1950 s. Most—but not all—of these studies have found longevity-related benefits associated with water hardness [3,6]. Interpretations of such data can be complicated when the geographic area of interest is too small to allow associations to be detected, or when monolithic water consumption is assumed for a wider geographical region with larger numbers of inhabitants without due consideration to the relevant water sources [3,7].

Scientific knowledge on water hardness and longevity would be enhanced from a comprehensive “lithosphere-to-lip” evaluation in an area of sufficient environmental variation and population homogeneity. The Amami islands—an island chain in the south-west of the Japanese archipelago—represent a promising region for such an evaluation. These islands are geologically well surveyed and two of them, Amami-Oshima and Tokunoshima, were recently inscribed on UNESCO’s World Heritage List as natural heritage sites that are rich in biodata [8]. Of the islands we focus on here, the northernmost island (Amami-Oshima) lies on a Paleogene accretionary complex, whereas the three more southerly islands (Tokunoshima, Okinoerabu, and Yoron) are largely coralline. These southerly islands share a basement and surface geology characterized by the presence of marine sediments and coralline limestone and were formed in the late Miocene, although one of them (Tokunoshima) also possesses a granite bedrock region of volcanic origin [9,10,11]. Such differences in the bedrock across this island chain might be expected to produce differences in water hardness, and we were able to make a preliminary confirmation of regional differences in municipally reported water hardness values (from information in the public domain or by personal correspondence with municipal officials).

Beyond the reported variation in water hardness, the Amami Islands offer other advantages for “lithosphere-to-lip” evaluations. The islands are clustered within a narrow band longitudinally and latitudinally, share a near-identical subtropical climate, and are inhabited by an ethnically and socioeconomically homogenous population that is largely unaffected by inward migration. The Japanese population generally enjoys the longest average life expectancy in the world [12], suggesting a lifelong exposure crucial to evaluating the influence of environmental factors. The Amami islanders appear even more longevous than the national population as a whole: the number of centenarians per 10,000 people (centenarian rate) across these islands is 14.9, versus the national figure of 5.62. Interestingly, Japan is generally a “soft water” country in which ground and surface water are blended to provide local drinking water; however, in the Amami islands, drinking water is drawn from groundwater. The geological variation in the Amami islands is known to drive variation in the local ecosphere (in terms of speciation and biodiversity), and it thus seems reasonable that differences may be similarly produced within the human population. Accordingly, we hypothesized that the variation in basement geology across the Amami islands would indirectly exert a differential effect on human longevity through differences in water hardness that stem from bedrock composition.

Investigating hypotheses related to the influence of drinking water hardness entails a degree of urgency at the current moment in time. With the marked rise in drinking bottled water and the drift of the population away from rural and island areas, it may be difficult to evaluate the effects of environmental factors on such local populations in future generations. However, drinking water is a key issue for the global community, and with rising sea levels, an increasing amount of the population will have to rely on remote water sources. With such prospective developments, it is vital for the scientific community to collect epidemiological data on the effects of local drinking water while such collection is still possible.

Against this background, we investigated the correlations between limestone bedrock occupancy and water hardness and between water hardness and the centenarian rate across the four above-stated Amami islands (encompassing 11 municipalities). We also evaluated other geographical and meteorological characteristics of these islands for their potential as confounders.

## 2. Materials and Methods

### 2.1. Regional Settings

**Figure 1:**
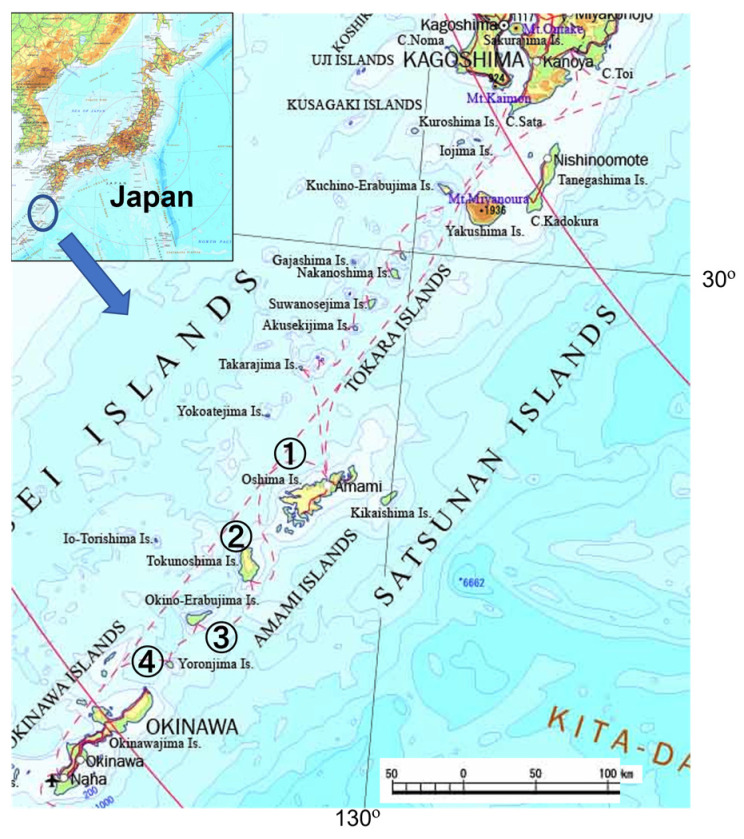
Map locations of the four Amami islands (1: Amami-Oshima; 2: Tokunoshima; 3: Okinoerabu; 4: Yoron). This map is a reproduction of the Digital Map published by Geospatial Information Authority of Japan.

We selected four islands of interest in this study from the Amami islands in Kagoshima prefecture, south-western Japan, as shown Figure 1. These islands were Amami-Oshima (28.2678° N, 129.3621° E), Tokunoshima (27.7904° N, 128.9668° E), Okinoerabu (27.3662° N, 128.6005° E), and Yoron (27.0426° N, 128.4324° E) [13]. These islands encompass 11 municipalities, which were used in data collection and analysis, as outlined below.

Amami-Oshima: Amami city, Tatsugo, Yamato, and Setouchi;

Tokunoshima: Amagi, Isen, and Tokunoshima Town;

Okinoerabu: Wadomari and China;

Yoron: unitary municipality of Yoron.

We targeted the four islands for analyses of the limestone bedrock occupancy, drinking water hardness, and longevity. Another member of this island chain, Kikaijima (28°19′35″ N 129°58′27″ E), was not included in water hardness evaluations because no relevant municipal data from this island were obtained in our preliminary investigation.

The population of the Amami islands has been little affected by inward migration, and agricultural occupations represent the largest single employment category, indicating that inhabitants have tended to work and reside in the same area for long periods.

The drinking water supply in each island is drawn from the groundwater. Local residents may draw water from their own private wells and/or obtain drawn water supplied by the local municipality.

### 2.2. Calculation of the Bedrock Occupancy (%)

The limestone bedrock occupancy was calculated as a percentage, based on a software-assisted visualization of the rock type distribution across the four islands of interest [10,14]. The procedure for this calculation was as outlined below.

**Figure 2:**
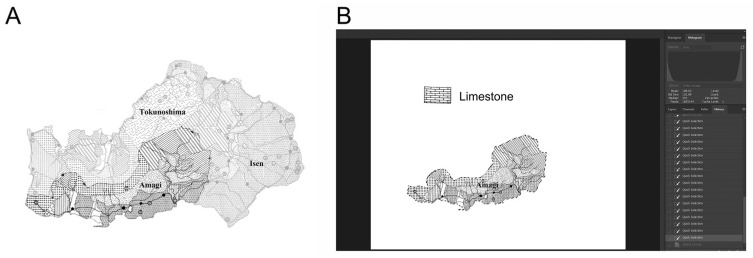
Image-based calculation of limestone bedrock occupancy (%) by municipality (representative image: Amagi municipality, Tokunoshima). The calculation was performed as an evaluation of pixelation within an active area in a Photoshop session. (**A**) The municipality of interest was targeted for a deeper, color-intensity software setting to make it visually distinct from the rest of the island. (**B**) The municipality of interest was then isolated by cropping, and limestone bedrock occupancy was calculated as a percentage of pixels corresponding to limestone versus the total number of pixels.

A cartographical image of each island with municipal boundaries superimposed over graphic representations of the bedrock was retrieved from the reference geological survey report [10] and converted for pixelation using ABBY FineReader 15 (Abbyy, Milpitas, CA, USA). The converted image was loaded into the image manipulation window of Photoshop Elements 2019 (Adobe, San Jose, CA, USA) and cropped from its white-space background using the software’s Lasso tool. The image of the island thus became the active layer in the Photoshop session, with different pixels corresponding to the geological characterization as indicated by the shading in the original survey report. The number of pixels corresponding to the graphic representation of limestone was calculated as a percentage against the total number of pixels in the active area (which corresponded to the whole island). This calculation was performed twice, and the mean of the two calculations was adopted as the limestone bedrock occupancy (%) value for the relevant island. To perform calculations at the municipality level, the Lasso tool was used again to separate the municipality of interest from the rest of the island; the intensity setting for the municipality was deepened to make it more visually distinct, and it was isolated by cropping it from the image of the island, discarding the unwanted municipalities. The number of pixels corresponding to the graphic representation of limestone was calculated as a percentage against the total number of pixels in the active area (which corresponded to the municipality). This calculation was performed twice, and the mean of the two calculations was adopted as the limestone bedrock occupancy (%) value for the relevant municipality. The calculation method is illustrated using the Amagi municipality on Tokunoshima as a representative example in Figure 2.

### 2.3. Drinking Water Sample Collection and Analyses

We collected drinking water from wells and water faucets at several different locations in the 11 above-stated municipalities across the four islands of interest. The calcium and magnesium ion concentrations of the collected water samples were then measured in an analysis commissioned to a specialist biochemical testing laboratory (Biken Co., Ltd., Kagoshima, Japan). The obtained calcium and magnesium ion concentrations are expressed as molar equivalents of calcium carbonate (CaCO_3_), based on the following formula: [CaCO_3_] = 2.5 × [Ca^2+^] + 4.1 × [Mg^2+^] (the respective molar masses of CaCO_3_, Ca, and Mg are 12, 16, 100.1, 40.1, and 24.3 g/mol; the respective molar ratios for Ca and Mg versus CaCO_3_ are thus 2.5 and 4.1). The measured calcium and magnesium ion concentrations were adopted as the hardness value for each alkaline earth metal. Water hardness was calculated as the sum of the calcium and magnesium hardnesses, using the following formula: Water hardness (mg/L) = Calcium hardness (mg/L) + Magnesium hardness (mg/L).

### 2.4. Collection and Handling of Demographic Data

For each municipality, the total population, number of centenarians, and the centenarian rate (calculated as the number of centenarians per 10,000 population) were obtained from publicly available census data published by the Japanese Ministry of Health, Labour and Welfare in 2019 [15].

### 2.5. Statistical Analysis

The results are expressed as means. Spearman’s rank correlation analysis was used to evaluate the relationship between the limestone bedrock occupancy (%) and the drinking water hardness and the relationship between the drinking water hardness and the centenarian rate (Excel 2016, Microsoft, Redmond, WA, USA). *p* values < 0.05 were considered significant.

## 3. Results

### 3.1. Regional and Meteorological Characteristics across the Islands of Interest

Using data collected by the Kagoshima Prefecture Government (Kagoshima, Japan) [16] and the Japanese Meteorological Agency [17] between 1991 and 2020, we evaluated the regional and meteorological characteristics of the islands of interest. Our evaluation for each island encompassed its area, circumference, maximum elevation, and the average annual temperature, precipitation, and humidity, as shown in Table 1. Although the average temperature broadly increases as the latitude becomes more southerly (in a north-to-south order, from Amami-Oshima, to Tokunoshima, Okinoerabu, and Yoron), the difference between the highest and lowest average temperatures is only 1.2 °C, making any temperature-related effect on relative longevity across the island chain unlikely. The annual precipitation increases as the latitude becomes more northerly and the humidity varies by only 1% where data are available.

### 3.2. Limestone Bedrock Occupancy (%) and Drinking Water Hardness by Island Municipality

Each island has a well-characterized surface and basement geology [10]. The key differences are that the three southerly islands of interest are coral reef islands in which marine sediment and coralline limestone dominate, whereas the northernmost island of interest has sandstone, slate, shale, quartzite, tuff, and granite in its bedrock. One of the coral reef islands, Tokunoshima, has northern regions of granite, although its limestone-rich southern region has been well documented. The limestone bedrock occupancy (%) and drinking water hardness are shown for the four islands of interest by municipality in Table 2. In the northernmost island of interest, Amami-Oshima, limestone was detected in only one of the four municipalities (Amami city), and that occupancy figure was very low (0.2%). Across the other islands of interest, the Yoron unitary municipality (82.9%) and the Isen municipality (86.5%) on Tokunoshima showed the highest limestone bedrock occupancy.

**Figure 3:**
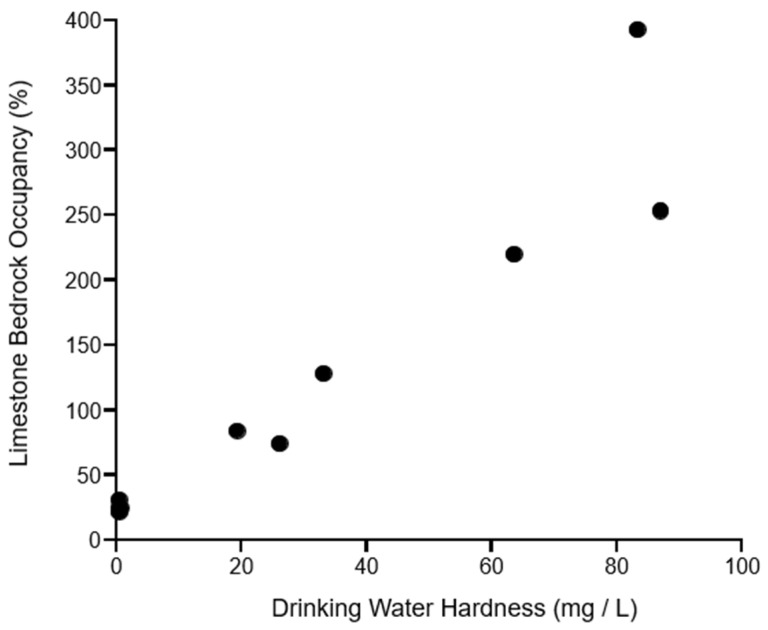
Relationship between limestone bedrock occupancy (LBO) and average of drinking water hardness (DWH) by island municipality. Black dots (n = 11) are plotted from municipal LBOs and DWHs.

The relationship between the limestone bedrock occupancy and the drinking water hardness is presented graphically in Figure 3. A Spearman’s rank correlation coefficient of 0.99 indicates a strong positive correlation (*p* < 0.01).

### 3.3. Centenarian Rates across Four Amami Islands by Municipality

The total population, number of centenarians, and the centenarian rate are shown by municipality for the four islands of interest in Table 2.

Compared by island, Yoron demonstrated the highest centenarian rate (29.7), followed by Tokunoshima (20.1) and then Okinoerabu (17.0), with Amami-Oshima showing the lowest rate (11.7), yielding a threefold difference between the lowest and highest figures.

Compared by municipality, the unitary Yoron municipality demonstrated the highest centenarian rate (29.7), and Tokunoshima’s Isen municipality showed the second highest centenarian rate (29.2).

### 3.4. Relationship between Centenarian Rate and Drinking Water Hardness

**Figure 4:**
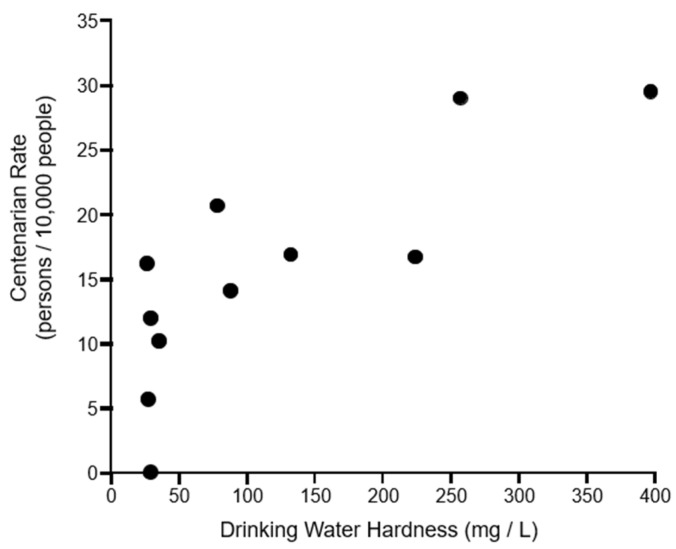
Relationship between centenarian rate (CR) and average of drinking water hardness (DWH) across four Amami islands by municipality. Black dots (n = 11) are plotted from municipal CRs and DWHs.

The relationship between the centenarian rate and the average drinking water hardness is presented graphically in Figure 4. A Spearman’s rank correlation coefficient of 0.84 indicates a strong positive correlation (*p* < 0.01).

## 4. Discussion

To the authors’ knowledge, this is the first study to geologically contextualize a potential effect of water hardness on longevity in a homogenous population in a small geographical area. Our study focuses on the Amami islands in the south-west of the Japanese archipelago, and we investigated relationships between the limestone bedrock occupancy, a major geological characteristic; water hardness; and the centenarian rate, an indicator of population longevity.

Regarding the major findings of this study, we found evidence to suggest that geology may drive a beneficial, water-hardness-related effect on the centenarian rate. Put crudely, we found that residents of areas rich in coralline limestone drink harder water and live longer. As might be expected, the water was harder in these limestone-rich areas, and we attribute this hardening to the leeching of cations from the calcium carbonate layer that the coralline limestone represents. In areas where the municipal water supply was drawn from limestone-hardened groundwater, the centenarian rate was higher, echoing the findings of increased longevity in areas of greater water hardness reported in many previous studies [6].

The geologically driven relationship between water hardness and longevity was apparent at the island level, with higher centenarian rates and water hardness values found across the three southerly coral reef islands than the northernmost island, which lies on a bedrock of sandstone, slate, shale, quartzite, tuff, and granite. The division between the southerly islands and the northernmost island appears to resemble (to a certain extent) a Wallacean biogeographical boundary. Differences in the ecosystem and speciation are already known to exist between the two sides of this divide, and the similarly geographically contrasting centenarian rates suggest that the biogeography is also driving the biogerontology for the human population here.

Our findings extend to below the island level, to more granular comparisons between the 11 municipalities across the four islands of interest. By municipality, the limestone bedrock occupancy (%) was very strongly correlated with water hardness (0.99), and water hardness was very strongly correlated with the centenarian rate (0.84). Notably, the two municipalities in which limestone occupied more than 80% of bedrock (Yoron and Isen in Tokunoshima) showed the highest centenarian rates (at 29.2 and 29.7, respectively); the difference between the rates in these municipalities and the rate in the next most longevous municipality rate is approximately double the national centenarian rate in itself (a difference of 8.3 versus a rate of 5.16 for Japan as a whole).

The island of Tokunoshima—and its limestone-rich municipality of Isen—yielded some of the most compelling evidence yet for an environmentally driven effect of water hardness on longevity. The Isen municipality showed clear differences relative to the other two more northerly municipalities (Tokunoshima Town and Amagi) on this island for the limestone bedrock occupancy (85.6% vs. 18.8% and 25.7%), water hardness (254 mg/mL vs. 76 and 86 mg/mL), and centenarian rate (29.2 vs. 20.9 and 14.3). These findings are important because they underline the strong correlations we found, and they are crucial for interpreting other meteorological and geographical data for the islands of interest. The precipitation increases and the average temperature decreases from south to north across the Amami islands; these parameters demonstrated a correlation with the centenarian rate (albeit a much weaker correlation than the water hardness). Considering the north–south pattern in Tokunoshima, we can largely attribute the precipitation and average temperature results to a latitude-dependent phenomena that is primarily geologically (not meteorologically) driven.

Our findings contribute to the accumulating evidence on the longevity-promoting effects of hard water. Recent examples of such research include studies in China [18] and Costa Rica [19] on longevity, and in Sardinia on protective effects against cardiovascular mortality [20]. In those studies, areas were not specifically geologically characterized; however, our study demonstrated how the limestone bedrock occupancy is related to the hardness of drinking water, which is in turn related to the centenarian rate across the 11 constituent municipalities of our four surveyed islands. Interestingly, water hardness values are reportedly associated with reduced cardiovascular mortality up to a maximum of 150–200 mg/L [21], whereas the water hardness values in our study appeared to be associated with the centenarian rate up to 250–400 mg/L.

Much of the biological plausibility of these results is based on scientific knowledge about the roles of calcium and magnesium in the body. Calcium is the most abundant metal in the human body and plays a vital role in physiological and biochemical processes in a range of organisms and cells through its involvement in signal transduction pathways, where its ions act as a second messenger.

Magnesium is the fourth most abundant metal in the human body and the second most abundant metal in the cells. Moreover, it is a biologically active mineral with a role as a cofactor in approximately three hundred enzymatic reactions in the human body [22]. Plasma and dietary magnesium levels are reportedly inversely associated with cardiovascular disease risk factors such as obesity, hypertension, dyslipidemia, type 2 diabetes, and coronary heart and cerebrovascular diseases [23,24,25]. Our group has also reported that higher 24 h urinary magnesium/creatinine ratios were associated with lower cardiovascular disease risk factor values [26,27]. Higher magnesium concentrations increase nitric oxide production from vascular endothelial cells [28,29], whereas lower concentrations of magnesium ions enhance adrenoceptor-related contractile responses via increased calcium influx [30,31]. Magnesium may have a role in preventing hypertension and cardiovascular disease.

Doubts exist regarding the optimum levels of magnesium and calcium in drinking water and the interplay between them. Some researchers have suggested that hard-water-derived magnesium has a beneficial effect but that calcium does not, or that calcium may even interfere with the beneficial effect of magnesium [6]. Establishing the optimal ratio of magnesium to calcium in hard water is beyond the scope of the present study; however, we believe that drinking water with a calcium-to-magnesium ratio determined by the presence of these two minerals in coralline limestone appears not to disrupt the beneficial effect of hard water.

This study possesses the inherent limitations of an ecological study. We have no data on the individual centurions living in the four islands of interest and thus cannot know their individual water-drinking histories. The potential for ecological fallacy can never be eliminated in this type of study, and it is difficult to control for confounding (even though we evaluated some potential confounders and found none of them to be influencing factors). Although our findings cannot not be taken as proof of causality, they are useful for hypothesis generation. Among Hill’s criteria for causation for epidemiological evaluations, two criteria (plausibility and specificity) may be met—in part—by our findings. It is biologically plausible that the calcium and magnesium absorbed into the body from drinking water have a preventative effect against heart disease. A degree of specificity is provided by our findings in one island: Tokunoshima. This island has a circumference of barely 84 km and an area of less than 250 km^2^. The greater centenarian rate in the one municipality—distinguished by the coralline limestone in its basement geology—on this small island thus appears to be a specific finding. These are reasons for believing that the hypothesis generated in this study is a promising one, but the limitations of the study prevent us from drawing conclusions beyond that.

Furthermore, our study was limited to an assessment of environmentally driven influences on longevity, as indicated by the centenarian rate. Although many broader, related questions in this study remain unanswered, our results suggest that the Amami Islands are well suited for environmentally contextualized epidemiological studies. The centenarian rate has been validated as a parameter for studying environmental correlates in the neighboring Okinawa islands [32]. In our study population, there was a demonstrable association with longevity overall, and similar populations could be targeted for research on other related questions. For example, the correlations we found in the population overall also applied to women, but the number of men we evaluated was not sufficient to demonstrate whether there was a gender difference. While many reports have focused on a protective effect against cardiovascular mortality, hard water may also enhance longevity by protecting against gastric cancer [33]. This study has generated useful evidence on the water hardness and longevity in the Amami islands, suggesting that these island populations could be targeted in other retrospective studies in this area. This could be of key importance since the sudden explosion of bottled water consumption and the upheaval in human migration may otherwise make future epidemiological and comparative studies difficult.

## 5. Conclusions

Based on the comparisons between demographically similar populations on Japanese islands with groundwater-based water supplies but differing geologies in this study, we hypothesize a potentially beneficial effect of hard water on longevity when that water is drawn from coralline limestone. Water hardness is just one among many factors that may influence life expectancy, but our findings are highly relevant to current debates about global access to drinking water and provide a basis for future research questions addressing the health benefits of mineral-rich water.

## Figures and Tables

**Table 1:** Regional and meteorological characteristics of four Amami islands.

Island	Area	Peak	Annual Average (1991–2020)			
	(km^2^)	(m)	Temp (°C)	PPT (mm)	AWS (m/s)	HMD (%)
Amami-Oshima	712.41	694	21.8	2935.7	2.6	74
Tokunoshima	247.85	645	21.8	1987.4	3.1	no data
Okinoerabu	93.65	240	22.6	1856.7	5.7	75
Yoron	20.56	97	22.9	1798.1	5.2	no data

Peak: maximum elevation; PPT: precipitation; AWS: average wind speed; HMD: humidity.

**Table 2:** Limestone bedrock occupancy (LBO), average of drinking water hardness (DWH) and centenarian rates in four Amami islands, by municipality (as of 1 October 2018).

No *	Municipalities	LBO	DWH	Total Population			No. of Centenarians			Centenarian Rate		
		(%)		Male	Female	Total	Male	Female	Total	Male	Female	Total
1	Amami	0.2	27	19,662	22,031	41,693	4	47	51	2.0	21.3	12.2
	Tatsugo	0	33	2770	3011	5781	0	6	6	0	19.9	10.4
	Yamato	0	27	703	727	1430	0	0	0	0	0	0
	Uken	0	25	803	882	1685	0	1	1	0	11.3	5.9
	Setouchi	0	24	4100	4456	8556	3	11	14	7.3	24.7	16.4
2	Amagi	25.7	76	2898	2840	5738	0	12	12	0	42.3	20.9
	Isen	86.5	255	3095	3076	6171	2	16	18	6.5	52.0	29.2
	Tokunoshima	18.8	86	5148	5333	10,481	2	13	15	3.9	24.4	14.3
3	Wadomari	32.7	130	3180	3262	6442	0	11	11	0	33.7	17.1
	China	63.2	222	2983	2937	5920	0	10	10	0	34.0	16.9
4	Yoron	82.9	395	2441	2615	5056	1	14	15	4.1	53.5	29.7

* Number indicates each island as shown in Figure 1. (1 = Amami-Oshima; 2 = Tokunoshima; 3 = Okinoerabu; 4 = Yoron). Centenarian rate is shown as number/10,000 population.

## Data Availability

Publicly available datasets were analyzed in this study. This data can be found here: http://hdl.handle.net/10232/2269 [10] for the geological survey data, and https://www.mhlw.go.jp/content/12304250/000547374.pdf [15] for the demographic data (centenarian rates).
